# Comparison of pregnancy outcomes between GnRH antagonist protocol with freeze-all strategy and long-acting GnRH agonist protocol in women with adenomyosis undergoing IVF/ICSI: a propensity-score matching analysis

**DOI:** 10.1186/s12884-022-05276-9

**Published:** 2022-12-17

**Authors:** Liting Zhang, He Cai, Xitong Liu, Yao Xiong, Xiaoling Liang, Juanzi Shi

**Affiliations:** The Assisted Reproduction Center, Northwest Women’s and Children’s Hospital, Affiliated With Xi’an Jiaotong University, Xi’an, China

**Keywords:** Adenomyosis, GnRH agonist, GnRH antagonist, IVF, Pregnancy outcomes

## Abstract

**Background:**

Plenty of studies explored the most optimal treatment protocol for infertile women with adenomyosis in in-vitro fertilization (IVF) /intracytoplasmic sperm injection (ICSI), however, there is still no consensus on which treatment protocol is ideal for these women at present. So, we conducted this study comparing the pregnancy outcomes in infertile women with ultrasound-diagnosed adenomyosis who underwent GnRH antagonist protocol with freeze-all strategy or long-acting GnRH agonist protocol.

**Methods:**

This was a retrospective cohort study and a propensity-score matching (PSM) analysis including 282 women diagnosed with adenomyosis undergoing their first IVF/ICSI cycle from January 2016 to July 2021 at the Assisted Reproduction Center, Northwest Women’s and Children’s Hospital, China. The patients were divided into two groups: the GnRH antagonist protocol with freeze-all strategy (*n* = 168) and the long-acting GnRH agonist protocol with fresh embryo transfer (*n* = 114) according their treatment protocols. The primary outcome was live birth rate. Cumulative live birth rate was also calculated.

**Results:**

After adjusting for confounders, clinical pregnancy rate (49.40% vs 64.04%; odds ratio (OR) 1.33; 95% confidence interval (CI) 0.70 to 2.37; *P* = 0.358), live birth rate (36.90% vs 45.61%; OR 1.10; 95% CI 0.61 to 2.00, *P* = 0.753) and cumulative live birth rate (51.79% vs 64.04%; OR 1.01; 95% CI 0.49 to 1.74, *P* = 0.796) were not significantly different between the GnRH antagonist protocol with freeze-all strategy and long-acting GnRH agonist protocol. Similar results were conducted in PSM analysis with clinical pregnancy rate (46.48% vs 60.56%; OR 1.33; 95% CI 0.76 to 2.34; *P* = 0.321), live birth rate (32.39% vs 45.07%; OR 1.31; 95% CI 0.63 to 2.72, *P* = 0.463) and cumulative live birth rate (54.90% vs 60.60%; OR 1.01; 95% CI 0.59 to 1.74, *P* = 0.958).

**Conclusions:**

For infertile women with adenomyosis, these two treatment protocols resulted in similar pregnancy outcomes. Larger, prospective studies are needed in the future.

## Background

Adenomyosis is a common gynecological disease in women of late childbearing age characterized by the existence of endometrial glands and stroma in the myometrium and impacts women’ s life quality [1]. With more women postponing pregnancy and the development of radiography methods such as 2D/3D transvaginal ultrasonography [2] and magnetic resonance imaging (MRI) [3], clinicians pay more and more attention to the impact of adenomyosis on infertility.

Studies reported that adenomyosis had negative effects to women’s fertility by reducing implantation rate, clinical pregnancy rate, live birth rate and increasing miscarriage rate [4–6]. Moreover, women diagnosed with adenomyosis have more obstetric complications such as premature rupture of membrane, preeclampsia and so on [7].

Recently, plenty of studies devote to explore the ideal treatment protocol to infertile women with adenomyosis in in-vitro fertilization (IVF) /intracytoplasmic sperm injection (ICSI) involving controlled ovarian hyperstimulation (COH) protocols, embryo transfer and pretreatment before embryo transfer [8]. However, there is still no consensus on which treatment protocol is the most optimal for these women.

Gonadotropin-releasing hormone (GnRH) agonist is not only used in COH protocol but also in the therapy of adenomyosis and endometriosis. Several studies support that in infertile women with adenomyosis, long-acting GnRH agonist protocol achieved better pregnancy outcomes after fresh embryo transfer [9, 10], with potential underlying mechanism, such as decreased expression of cytochrome P450 in adenomyosis lesions [11], down-regulation of circulating estrogen levels by inhibiting hypothalamic-pituitary axis, improved microenvironment and follicular quality [12], and ameliorative endometrial receptivity by up-regulating Hoxa10, Hoxa11, Lif and integrinβ3 [13]. However, due to possible excessive suppression of hypothalamic-pituitary axis and following poor ovarian response [14], long-acting GnRH agonist protocol is not suitable for all women, especially those with poor ovarian reserve. Recently, the GnRH antagonist protocol is widely used due to the shorter treatment duration, the lower dose of gonadotropin, and higher patient compliance [15].

During fresh embryo transfer cycles, the supra-physiological elevation of estrogen levels in the COH procedure are deleterious to both embryos and endometrium [16]. Moreover, the significantly elevated estrogen levels might aggravate adenomyosis owing to its estrogen-dependent nature. A multicenter, randomized controlled trial (RCT) found that frozen embryo transfer (FET) achieved a higher live birth rate and a lower pregnancy loss rate than fresh embryo transfer in infertile women with polycystic ovary syndrome (PCOS) [17]. However, this finding was not confirmed in ovulatory women [18]. Furthermore, there are only few studies focused on the outcome differences between fresh embryo transfer cycles and FET cycles in women with adenomyosis [19].

To gain more insight of the most appropriate treatment protocol for infertile women with adenomyosis, we conducted this study comparing the pregnancy outcomes in infertile women with ultrasound-diagnosed adenomyosis in two treatment protocols, one is GnRH antagonist protocol with freeze-all strategy, the other is long-acting GnRH agonist protocol with fresh embryo transfer.

## Method

### Study design and population

This retrospective cohort study included women with adenomyosis who underwent their first IVF/ICSI cycle at the Northwest Women’s and Children’s Hospital from January 2016 to July 2021.

Women with adenomyosis who underwent their first IVF/ICSI cycle with either GnRH antagonist protocol and freeze-all strategy (group A), or long-acting GnRH agonist protocol with fresh embryo transfer (group B) were enrolled. For women in GnRH antagonist protocol, they all received their first FET with hormone replacement therapy (HRT) following long-acting GnRH agonist pretreatment, but no specific restriction about GnRH agonist pretreatment in following FET. For women in long-acting GnRH agonist protocol, they all received fresh embryo transfer.

**Fig. 1:**
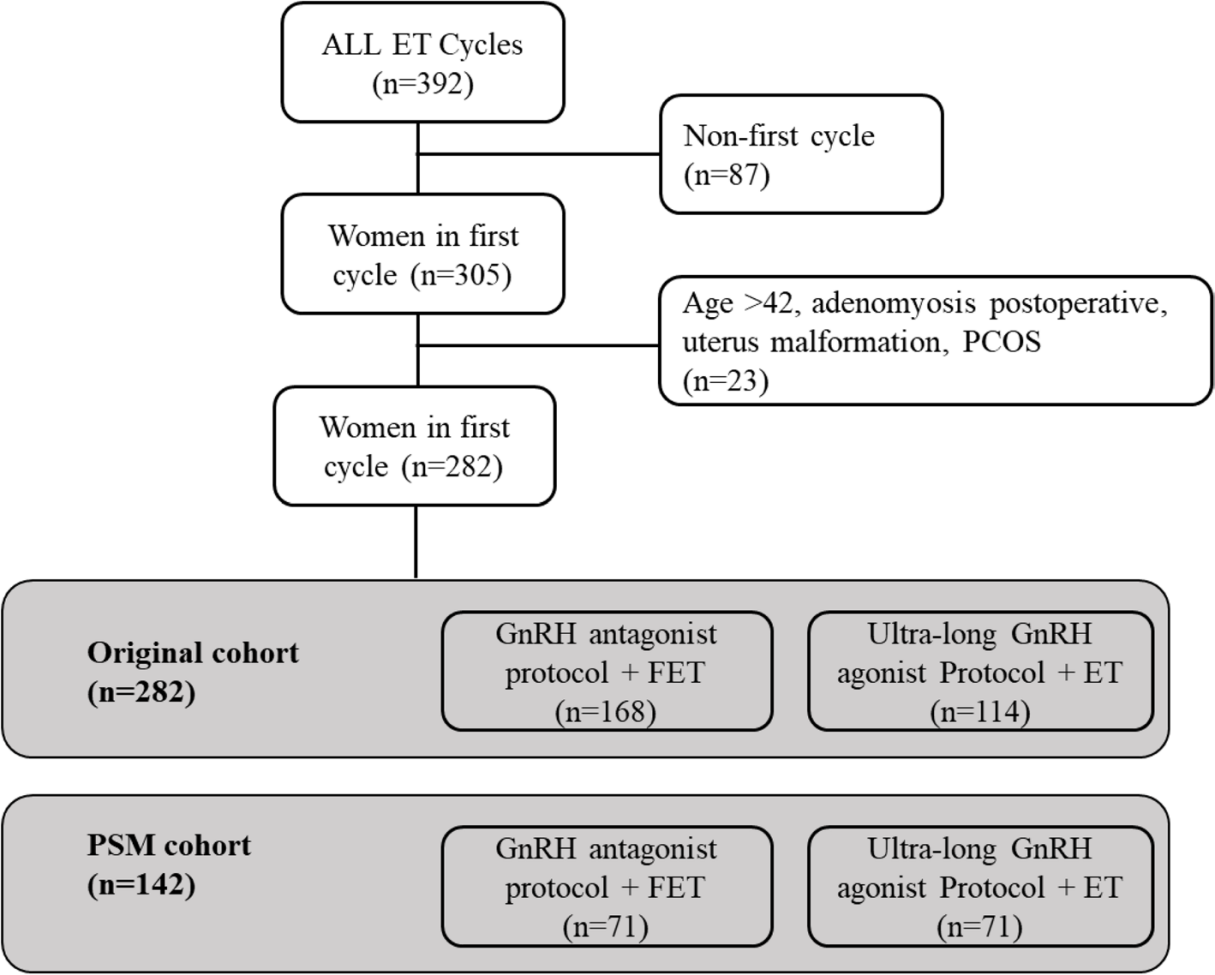
The study flow chart

The data was collected from electronic medical record system. Women aged above 42 years old, previous surgery for adenomyosis, uterine malformation, untreated intrauterine lesions, untreated hydrosalpinx, PCOS, uncontrolled systematic diseases and preimplantation genetic testing (PGT) cycles were excluded from this study. In total, 282 women were included, 168 women received GnRH antagonist protocol and freeze-all strategy and 114 women received long-acting GnRH agonist protocol (Fig. 1).

All women had a baseline transvaginal ultrasound prior to commencement of IVF/ICSI. Adenomyosis diagnosis was based on the standard radiological criteria: (1) enlargement of the uterine corpus, (2) asymmetrically thickened myometrium of uterine walls, (3) poor definition of the junctional zone, (4) heterogeneity of the myometrium or hypoechoic striations, and (5) sub-endometrial myometrial cysts [20]. The basal uterine volume was calculated by baseline transvaginal ultrasound screening using a geometric formula: long diameter × width diameter × anteroposterior diameter × π/6 [21].

### IVF/ICSI treatment protocols

The appropriate COH protocol was offered to women based on their age, body mass index (BMI), antral follicle count (AFC) and menstrual cycle.

In women received GnRH-antagonist protocol, COH with daily dosage of gonadotropin (150–300 IU) was initiated on day 2–4 of menstrual cycle, and the dosage was adjusted according to women’s age, BMI, and ovarian reserve. GnRH antagonist 0.25 mg /day was given when at least one follicle was ≥12 -14 mm in mean diameter until the trigger day (including the trigger day). This group of women received freeze-all strategy and following FET.

The women with long-acting GnRH-agonist protocol underwent long-acting GnRH agonist (3.75 mg) subcutaneous injection starting on day 2–5 of menstrual cycle for one or more times. The size of uterus was measured 30 days after each injection, if the anteroposterior diameter exceeded 70 mm, long-acting GnRH agonist would be injected a second time. After 30 days of the last long-acting GnRH agonist injection, once endometrium thickness ≤ 5 mm, serum estradiol≤50 pg/mL, and LH ≤5 IU/L were confirmed, COH was started with 150–300 IU/day gonadotropin according to women’s age, weight, and ovarian reserve. Women in this group all received fresh embryo transfer.

### GnRH agonist pretreatment and HRT endometrial preparation in FET

Women received one or more times of 3.75 mg long-acting GnRH agonist on day 2 of the cycle after an ultrasound scan confirmed ovarian quiescence and the presence of a thin endometrium (< 5 mm). After 28 to 30 days following the last injection, estradiol valerate (Progynova; Bayer Schering Pharma AG, Berlin, Germany) was administered orally at 4 to 6 mg daily. Approximately 10 to 12 days later, vaginal progesterone was administered to achieve endometrial transformation as soon as the endometrial thickness reached 7 mm and the serum progesterone level was < 1.5 ng/mL. FET was then performed 3 days (cleavage-stage embryos) or 5 days (blastocysts) after progesterone therapy.

### Study outcomes

The primary outcome was live birth rate defined as delivery of neonate ≥28 week’s gestation with heart beat and breath after first embryo transfer. Secondary outcomes were clinical pregnancy rate, miscarriage rate and preterm delivery rate of first embryo transfer and cumulative live birth rate (CLBR). Clinical pregnancy was defined as the presence of at least one intrauterine gestational sac at approximately 6-week gestation ultrasound. Miscarriage was defined as fetal delivery at < 28 weeks of gestational age. Preterm delivery was defined as fetal delivery at ≥28 weeks but < 37 weeks of gestational age. All above outcomes were calculated for each patient. The CLBR was calculated by including the first live birth attributable to IVF/ICSI cycle within 12 months after COH, which is the numerator, and the denominator was defined as all women.

### Statistical method

Propensity-score matching (PSM) was performed to adjust for confounding factors related to achieving pregnancy outcomes, the variables in the PSM included age, BMI, AFC, infertility type and infertility duration, which allowed a part of women in two groups can be matched reciprocally with similar characteristics. To optimize the precision of the study, the match was conducted in a 1:1 matching ratio without replacement, and with a caliper width equal to 0.01 of the standard deviation of the logit of the propensity score. Standardized differences were estimated.

Data were expressed as mean ± standard deviation (SD), Median (Q1-Q3) or n (%). Descriptive data were compared by Student’s T, Mann–Whitney U, Chi-squared or Fishers’ exact tests when appropriate in original cohort, and paired paired t test, Wilcoxon signed-rank test, and McNemar’s test was used in PSM cohort. Logistic regression was used to compare clinical pregnancy rate, live birth rate after adjusting for several confounders, and conditional logistic regression was used in PSM cohort. We selected these confounders on the basis of their associations with the outcomes of interest or a change in effect estimate of more than 10%. Data were analyzed using the statistical packages R (The R Foundation; https://www.r-project.org; version3.4.3) and Empower (R) (www.empowerstates.com, X&Y solutions, inc. Boston, Massachusetts). *P* < 0.05 was considered to be significant.

## Results

### Baseline characteristics

In this study, 282 women with adenomyosis were recruited in original cohort and 142 women in PSM cohort. The baseline characteristics were shown in Table 1. In original cohort, women who received long-acting GnRH agonist protocol were apparently younger (*P* < 0.001) and had more basal AFC (P < 0.001) and smaller basal uterine volume (*P* = 0.002) compared to women in GnRH antagonist protocol. No significant differences were found in BMI, infertility duration, infertility type and co-occurring with endometriosis between two groups. After PSM, the baseline characteristics between two groups reached a well balance in age, BMI, basal AFC, infertility type and infertility duration with a standardized difference below 10%. However, in PSM cohort, women with long-acting GnRH agonist protocol still had smaller basal uterine volume (*P* = 0.023).

**Table 1:** Baseline characteristics before and after propensity score matching (PSM) between different treatment protocols

Variables	Original cohort			P	PSM cohort			P
	Group A (n = 168)	Group B (n = 114)	Standardized difference		Group A (*n* = 71)	Group B (n = 71)	Standardized difference	
Age (years)	33.62 ± 4.00	31.67 ± 3.64	0.51	< 0.001	32.30 ± 4.07	32.21 ± 3.85	0.02	0.892
BMI (kg/m2)	22.24 ± 3.12	22.85 ± 3.26	0.19	0.115	22.23 ± 2.79	22.18 ± 3.05	0.02	0.913
Basal AFC (n)	7.50 (5.00–10.00)	12.00 (9.00–16.00)	1.13	< 0.001	10.00 (7.00–13.00)	10.00 (7.00–12.00)	0.02	0.687
Infertility duration (years)	3.00 (2.00–4.00)	3.00 (2.00–5.00)	0.02	0.881	2.00 (2.00–4.00)	3.00 (2.00–5.00)	0.02	0.847
Infertility type (n, %)			0.19	0.120			0.06	0.839
Primary	77 (45.83%)	63 (55.26%)			39 (54.93%)	37 (52.11%)		
Secondary	91 (54.17%)	51 (44.74%)			32 (45.07%)	34 (47.89%)		
Basal uterine volume (cm^3^)	97.65(69.65–141.68)	72.58(52.32–109.96)	–	0.002	97.97 (71.85–133.77)	71.57 (50.61–124.65)	–	0.023
Co-occurring with endometriosis (n, %)	55 (32.74%)	27 (23.68%)	–	0.100	29 (40.85%)	22 (30.99%)	–	0.230

Note: Group A = GnRH antagonist protocol and freeze-all strategy; Group B = long-acting GnRH agonist protocol; PSM = propensity-score matching; BMI = body mass index; AFC = antral follicle count

### Treatment characteristics in IVF/ICSI

Treatment information in two groups were exhibited in Table 2. Before PSM, there was a statistically significant difference in stimulation duration (*P* < 0.001), the number of oocytes retrieved (P < 0.001), number of fertilization (*P* = 0.001), number of 2PN (*P* = 0.002), number of available embryos (*P* = 0.011), number of transferred high-quality embryos (*P* = 0.007) and embryo stage transferred (P = 0.002) between two groups. After PSM, women received long-acting GnRH agonist protocol had higher gonadotropin dosage (P = 0.001) and longer stimulation duration (*P* < 0.001) compared women with GnRH antagonist protocol. There were no statistically significant differences in other characteristics between the groups.

**Table 2:** Ovarian stimulation characteristics of two treatment protocols

	Original cohort		P	PSM cohort		P
	Group A (n = 168)	Group B (n = 114)		Group A (n = 71)	Group B (n = 71)	
Gonadotropin dosage (IU)	2639.36 ± 741.08	2697.37 ± 968.87	0.570	2406.69 ± 564.97	2930.28 ± 1040.40	0.001
Duration of stimulation (days)	9.77 ± 1.63	11.84 ± 2.98	< 0.001	9.49 ± 1.29	11.93 ± 3.05	< 0.001
Number of oocytes retrieved (n)	7.00 (4.00–10.00)	10.00 (7.00–12.75)	< 0.001	8.00 (6.00–12.50)	9.00 (6.00–12.00)	0.978
Fertilization type (n, %)			0.162			0.431
IVF	144 (85.71%)	88 (77.19%)		62 (87.32%)	57 (80.28%)	
ICSI	19 (11.31%)	22 (19.30%)		8 (11.27%)	11 (15.49%)	
IVF + ICSI	5 (2.98%)	4 (3.51%)		1 (1.41%)	3 (4.23%)	
Number of fertilization (n)	6.00 (4.00–8.00)	8.00 (5.00–10.00)	0.001	7.00 (5.00–10.50)	7.00 (5.00–10.00)	0.606
2PN(n)	4.00 (3.00–7.00)	6.00 (4.00–8.00)	0.002	6.00 (4.00–7.50)	5.00 (4.00–8.00)	0.766
Number of available embryos (n)	4.00 (3.00–6.00)	5.00 (3.00–7.00)	0.011	5.00 (3.00–700)	5.00 (2.50–700)	0.818
Number of high-quality embryos (n)	2.00 (1.00–4.00)	3.00 (2.00–5.00)	0.080	3.00 (2.00–5.00)	3.00 (2.00–5.00)	0.461
Number of transferred embryos (n, %)			0.420			0.263
1	96 (57.14%)	68 (59.65%)		49 (69.01%)	41 (57.75%)	
2	72 (42.86%)	45 (39.47%)		22 (30.99%)	29 (40.85%)	
3	0 (0.00%)	1 (0.88%)		0 (0.00%)	1 (1.41%)	
Number of transferred high-quality embryos (n, %)			0.007			0.009
0	69(41.07%)	39(34.21%)		30(42.25%)	24(33.80%)	
1	91(54.17%)	57(50.00%)		40(56.34%)	33(46.68%)	
2	8(4.76%)	18(15.79%)		1(1.41%)	14(19.72%)	
Embryo stage (n, %)			0.002			>0.99
Cleavage stage	99 (58.93%)	46 (40.35%)		32 (45.07%)	33 (46.48%)	
Blastocyst stage	69 (41.07%)	68 (59.65%)		39 (54.93%)	38 (53.52%)	

Note: Group A = GnRH antagonist protocol and freeze-all strategy; Group B = long-acting GnRH agonist protocol; PSM = propensity-score matching; IVF = in vitro fertilization; ICSI = intracytoplasmic sperm injection; 2PN = two pronuclear

### Pregnancy outcomes

The pregnancy outcomes were presented in Table 3. In original cohort, compared with women with GnRH antagonist protocol and freeze-all strategy, women using long-acting GnRH agonist protocol conducted higher clinical pregnancy rate (49.40% vs 64.04%), miscarriage rate (12.50% vs 17.54%), live birth rate (36.90% vs 45.61%), twin pregnancy rate (8.06% vs 19.23%), preterm delivery rate (8.93% vs 13.16%) and CLBR (51.79% vs 64.04%), but only clinical pregnancy rate (*P* = 0.015) and CLBR (*P* = 0.042) showed a significantly statistical difference. PSM cohort had similar pregnancy outcomes with original cohort, which showed that clinical pregnancy rate was 46.48% vs 60.56%, miscarriage rate was 14.08% vs 15.49%, live birth rate was 32.39% vs 45.07% and CLBR was 54.90% vs 60.60% between GnRH antagonist protocol and long-acting GnRH agonist protocol. However, all differences didn’t reach statistical difference in PSM cohort. The results of logistic regression and conditional logistic regression were presented in Table 4. After adjusting for potential confounders presented in Table 4, no difference was found in live birth between two groups after adjusting for covariates in original cohort (OR 1.10, 95%CI, 0.61 to 2.00, *P* = 0.753), after PSM (OR 1.39, 95%CI, 0.81 to 2.38, P = 0. 227) and after adjusting for covariates in PSM cohort (OR 1.31, 95%CI, 0.63 to 2.72, *P* = 0.463). Other pregnancy outcomes were not statistically different in regression analysis (Table 4).

**Table 3:** Pregnancy outcomes of two treatment protocols

	Original cohort		P	PSM cohort		P
	Group A (n = 168)	Group B (n = 114)		Group A (n = 71)	Group B (n = 71)	
Clinical pregnancy rate (n, %)	83 (49.40%)	73 (64.04%)	0.015	33 (46.48%)	43 (60.56%)	0.130
Miscarriage rate (n, %)	21 (12.50%)	20 (17.54%)	0.238	10 (14.09%)	11 (15.49%)	0.813
Early miscarriage	17(10.12%)	17(14.91%)	0.225	8(11.27%)	10(14.08%)	0.796
Late miscarriage	4(2.38%)	3(2.63%)	0.894	2(2.81%)	1(1.41%)	0.560
Live birth rate (n, %)	62 (36.90%)	52 (45.61%)	0.144	23 (32.39%)	32 (45.07%)	0.168
Single/twin			0.079			0.214
Single (n, %)	57(91.94%)	42(80.77%)		20 (86.96%)	25 (78.12%)	
Twin (n, %)	5(8.06%)	10(19.23)		3 (13.04%)	7 (21.88%)	
Preterm delivery rate (n, %)	15 (8.93%)	15 (13.16%)	0.258	5 (7.04%)	11 (15.49%)	0.185
Cumulative live birth rate (n, %)	87/168(51.79%)	73/114(64.04%)	0.042	39/71(54.90%)	43/71(60.60%)	0.610

Note: Group A = GnRH antagonist protocol and freeze-all strategy; Group B = long-acting GnRH agonist protocol; PSM = propensity-score matching

**Table 4:** Multivariable logistic regression of pregnancy outcomes in different treatment protocols

Pregnancy outcomes	Original cohort				PSM cohort		
	Crude OR ^a^ (95% CI)		Adjusted OR ^b^ (95% CI)		Crude OR ^a^ (95% CI)		Adjusted OR ^c^ (95% CI)
Clinical pregnancy rate	1.82(1.12,2.97) ^d^		1.73(0.90,3.32)		1.30(0.83,2.05)		1.28(0.75,2.21)
Miscarriage rate	1.49(0.77,2.90)		1.85(0.73,4.71)		1.10(0.47,2.59)		1.27(0.43,3.73)
Live birth rate	1.43(0.88,2.33)		1.22(0.65,2.30)		1.39(0.81,2.38)		1.29(0.66,2.54)
Preterm delivery rate	1.55(0.72,3.30)		1.53(0.57,4.07)		2.20(0.76,6.33)		2.06(0.52,8.25)
Cumulative live birth rate	1.71(1.04,2.82) ^d^		1.17(0.58,2.38)		1.10(0.71,1.70)		0.98(0.58,1.67)

Note: Group A = GnRH antagonist protocol and freeze-all strategy; Group B = long-acting GnRH agonist protocol; PSM = propensity-score matching; OR = odds ratio; CI = confidence interval; BMI = body mass index; AFC = antral follicle count

^a^ No adjustments for covariates

^b^ Adjusted for age, BMI, AFC, basal uterine volume, number of transferred embryos, embryo stage, number of transferred high-quality embryos, number of available embryos

^c^ Adjusted for basal uterine volume, number of transferred embryos, embryo stage, number of transferred high-quality embryos, number of available embryos

^d^ *P* < 0.05

## Discussion

In this study, women receiving fresh embryo transfer after long-acting GnRH agonist protocol reached slightly higher clinical pregnancy rate, live birth rate and CLBR, also higher miscarriage rate and preterm delivery rate in both original cohort and PSM cohort. However, these differences were not statistically significant after adjusting confounders.

A plenty of studies tried to explore the most appropriate treatment protocol to infertile women with adenomyosis. A meta-analysis found that the long stimulation protocol had better outcomes compared to short stimulation protocol in pregnancy rate, live birth, and miscarriage in adenomyosis women [22]. Studies also showed that women with adenomyosis following the ultra-long GnRH agonist protocol have a better pregnancy outcomes than those following the long GnRH agonist protocol [9, 10]. Thalluri et al. demonstrated that following GnRH antagonist protocol, compared to infertile women without adenomyosis, women with adenomyosis had apparently lower clinical pregnancy rate [23]. Other studies support that GnRH agonist may improve the pregnancy outcomes of IVF/ICSI involving fresh embryo transfer or FET [24, 25]. These studies indicated that GnRH agonist treatment seems to get better pregnancy outcomes in women with adenomyosis.

Wu et al. found that FET following long-term GnRH agonist pretreatment had a higher live birth rate than fresh embryo transfer with a long or ultra-long GnRH agonist protocol [26]. Another study showed that vitrified-warmed embryo transfer achieved a higher singleton live birth rate and lower risk of preterm birth than fresh embryo transfer in women with adenomyosis [19], however, this study did not compare different COH protocols.

In contrast, our results showed that long-acting GnRH agonist pretreatment before FET in HRT cycle following GnRH antagonist protocol didn’t reach a higher clinical pregnancy rate, live birth rate and CLBR. Previous studies exhibited similar results, which found that long-acting GnRH agonist based on the HRT cycle may not increase the rate of clinical pregnancy or live birth [27]. Severity degree of adenomyosis is a quite important factor which impacts pregnancy outcomes. Research has shown that women with adenomyosis with larger uterine volume before FET might have a lower live birth rate and higher incidence of miscarriage [28]. In this study, women undergoing GnRH antagonist protocol had larger basal uterine volume in both original cohort and PSM cohort, which might partly contribute to the poorer pregnancy outcomes in this group.

There are several strengths of this study. Firstly, only women undergoing first IVF/ICSI cycle were included which avoided the selection bias of treatment protocol brought by multiple cycles. Secondly, We adopted internationally accepted criteria for the diagnosis of adenomyosis through transvaginal ultrasound scans.

There are limitations of this study. This is a retrospective study, therefore selection bias cannot be avoided. Moreover, we only used basal uterine volume to represent severity degree of adenomyosis. However, location, scope of adenomyosis lesion and whether endometrium is affected are related to pregnancy outcomes [29]. Furthermore, women in GnRH antagonist protocol were older and had less basal AFC than women in long-acting GnRH agonist protocol in this study, since clinicians wouldn’t choose long-acting GnRH agonist protocol for women with less basal AFC to avoid excessive ovarian suppression. PSM was conducted to control these baseline differences between two groups to achieve a relatively good balance.

## Conclusion

In conclusion, these two treatment protocols to infertile women with adenomyosis resulted in similar pregnancy outcomes. Larger, prospective studies with more detailed information about adenomyosis are needed to further evaluate the ideal treatment protocol among women with adenomyosis undergoing IVF/ICSI.

## Data Availability

The datasets used and/or analysed during the current study are available from the corresponding author on reasonable request.

## Abbreviations

IVF: in-vitro fertilization
ICSI: intracytoplasmic sperm injection
PSM: propensity-score matching
OR: odds ratio
CI: confidence interval
MRI: magnetic resonance imaging
COH: controlled ovarian hyperstimulation
GnRH: Gonadotropin-releasing hormone
RCT: randomized controlled trial
FET: frozen embryo transfer
PCOS: polycystic ovary syndrome
HRT: hormone replacement therapy
PGT: preimplantation genetic testing
BMI: body mass index
AFC: antral follicle count
CLBR: cumulative live birth rate.
